# Leaf litter from *Cynanchum auriculatum* Royle ex Wight leads to root rot outbreaks by *Fusarium solani*, hindering continuous cropping

**DOI:** 10.1093/femsec/fiae068

**Published:** 2024-04-29

**Authors:** Min Shen, Limeng Wu, Yanzhou Zhang, Ruiqiang You, Jiaxin Xiao, Yijun Kang

**Affiliations:** College of Life Sciences, Anhui Normal University, Wuhu, Anhui, 241000, China; Jiangsu Key Laboratory for Bioresources of Saline Soils, Yancheng Teachers University, Yancheng, Jiangsu, 224007, China; Jiangsu Key Laboratory for Bioresources of Saline Soils, Jiangsu Provincial Key Laboratory of Coastal Wetland Bioresources and Environmental Protection, Yancheng Teachers University, Yancheng, 224007, China; Jiangsu Key Laboratory for Bioresources of Saline Soils, Yancheng Teachers University, Yancheng, Jiangsu, 224007, China; Jiangsu Key Laboratory for Bioresources of Saline Soils, Yancheng Teachers University, Yancheng, Jiangsu, 224007, China; Jiangsu Key Laboratory for Bioresources of Saline Soils, Yancheng Teachers University, Yancheng, Jiangsu, 224007, China; College of Life Sciences, Anhui Normal University, Wuhu, Anhui, 241000, China; Jiangsu Key Laboratory for Bioresources of Saline Soils, Yancheng Teachers University, Yancheng, Jiangsu, 224007, China; Jiangsu Key Laboratory for Bioresources of Saline Soils, Jiangsu Provincial Key Laboratory of Coastal Wetland Bioresources and Environmental Protection, Yancheng Teachers University, Yancheng, 224007, China

**Keywords:** *Cynanchum auriculatum* Royle ex Wight, *Fusarium solani* D1, leaf litter, microbial community, phenolic acids

## Abstract

*Cynanchum auriculatum* Royle ex Wight (*CA*) is experiencing challenges with continuous cropping obstacle (CCO) due to soil-borne fungal pathogens. The leaf litter from *CA* is regularly incorporated into the soil after root harvesting, but the impact of this practice on pathogen outbreaks remains uncertain. In this study, a fungal strain D1, identified as *Fusarium solani*, was isolated and confirmed as a potential factor in CCO. Both leave extract (LE) and root extract (RE) were found to inhibit seed germination and the activities of plant defense-related enzymes. The combinations of extracts and D1 exacerbated these negative effects. Beyond promoting the proliferation of D1 in soil, the extracts also enhanced the hypha weight, spore number, and spore germination rate of D1. Compared to RE, LE exhibited a greater degree of promotion in the activities of pathogenesis-related enzymes in D1. Additionally, caffeic acid and ferulic acid were identified as potential active compounds. LE, particularly in combination with D1, induced a shift in the composition of fungal communities rather than bacterial communities. These findings indicate that the water extract of leaf litter stimulated the growth and proliferation of fungal strain D1, thereby augmenting its pathogenicity toward *CA* and ultimately contributing to the CCO process.

## Introduction

Continuous planting frequently leads to the suppression of seed germination and plant growth, a phenomenon referred to as the continuous cropping obstacle (CCO). This obstacle is observed in numerous plant species, particularly in medicinal plants like *Panax quinquefolius* L. (Dong et al. 2016, Tan et al. 2017, Liao et al. 2018, Li et al. 2020, 2021, Zhang et al. 2020), *Pogostemon cablin* (Zeng et al. 2020), *Angelica sinensis* (Xinhui 2010), and *Amomum villosum* (Wang et al. 2022). Additionally, CCO is also evident in *Cynanchum auriculatum* Royle ex Wight (*CA*), commonly known as “Binhai Baishouwu,” primarily found in Binhai county, Jiangsu Province, China (Jiang et al. 2011). Several studies have demonstrated the significant medicinal properties of *CA*, such as its antitumor, immunomodulatory, hepatoprotective, anti-inflammatory, and antidepressant activities (Chen et al. 2019). Consequently, *CA* cultivation is extensively practiced in China and Korea due to its substantial economic advantages (Kim et al. 2018). Nevertheless, the limited expansion of *CA* planting areas can be attributed to the decrease in *CA* yields caused by CCO (Chen et al. 2022b).

Various perspectives have been employed to investigate the mechanisms underlying CCO. Numerous studies have reported and widely acknowledged alterations in microbial structure and diversity, as well as the degradation of soil properties resulting from the accumulation of phenolic acids and other autotoxins (Dong et al. 2016, Tan et al. 2017, Bai et al. 2019, Li et al. 2020, Wang et al. 2020, 2022, Chen et al. 2022b). Chen et al. (2020) additionally highlighted that different phases of strawberry cultivation exhibit distinct changes in CCO. Building upon these findings, several strategies have been proposed and successfully implemented, including the addition of arbuscular mycorrhizal fungi and calcium (CUI et al. 2019), the utilization of microalgae (Feng et al. 2022), novel bioorganic fertilizer (Ling et al. 2014), and the adoption of stereoscopic cultivation (Liao et al. 2018) and intercropping systems (Zeng et al. 2020).

To date, no relevant reports pertaining to CCO in *CA* have been identified. Based on our field investigation, it has been observed that root rot disease is highly prevalent during the continuous cropping of *CA*. We also observed that that the aboveground components of *CA*, particularly the leaves, are left to fall to the ground without being harvested or utilized due to their unappealing taste to animals. Consequently, these components are subsequently incorporated into the ploughed layers after the roots have been harvested. Although leaf litter has been shown to contribute more to soil organic carbon than fine roots in certain plantations (Cao et al. 2020) and serves as a protective measure against runoff and erosion (Li et al. 2014), this practice does not align with the requirements of medicinal plants. A study demonstrated that the leachate derived from the stems and leaves of commonly used medicinal materials exhibited noteworthy inhibitory effects on the release of carbon, nitrogen, and phosphorus during litter decomposition, as well as on the activities of all seven types of soil enzymes (Yu-Peng et al. 2017). Given the substantial amount of leaf litter that accumulates in the soil during each planting season in *CA*, coupled with the presence of various allelopathic substances in the leaves of numerous medicinal plants (Basotra et al. 2005, Gupta 2016, Aniya et al. 2020), we propose the hypothesis that the inhibitory effects on *CA* growth are primarily attributed to the deposition of leaf litter rather than the roots extraction. Previous research has demonstrated the potential inhibitory impact of leaf litter on the growth of plants. Furthermore, the primary association between leaf litter and the plant growth index is attributed to alterations in the composition and diversity of soil microbial communities (Chen et al. 2022b), particularly characterized by an increase in pathogenic bacteria and a decrease in beneficial microorganisms (Pang et al. 2021, Wei-Ye et al. 2022). However, it is important to note that these findings were derived from high-throughput sequencing techniques, and there remains a dearth of evidence from pure culture experiments.

To mechanistically test our hypothesis that inhibitory effects on *CA* growth are primarily attributed to the deposition of leaf litter rather than the roots extraction, we contacted inoculation experiments with an isolated a significant fungal pathogen. Subsequently, we conducted a comparative analysis of the distinct impacts of root and leaf extracts on various indices pertaining to plant growth, soil quality, and pathogen activity. The outcomes of this investigation offer a theoretical foundation for elucidating the mechanism of CCO and enhancing the field management of *CA*.

## Materials and methods

### Isolation and identification of fungal pathogen causing root rot disease

On 22 May 2022, soil samples were obtained from the rhizosphere of *CA* plants afflicted with root rot disease in a planting base (117.71071 E, 39.0032 N) with a cultivation history of at least 15 years. To collect the soil, *CA* roots were vigorously shaken to dislodge loosely adhered soil particles. The dislodged soil was carefully separated and collected using gloved hands, and considered as rhizosphere soil. Subsequently, the soil sample was promptly transported to the laboratory at Yancheng Teachers University in a cooler box, ensuring a maximum time lapse of 12 h. The soil properties were assessed as follows: pH _(H2O)_ was determined to be 8.30 using a precalibrated glass electrode in a 1:2 soil/distilled water suspension (Mclean 2015). The total nitrogen content was measured to be 1.07 g/kg by using UV radiation digestion and subsequent oxidation with potassium persulfate in an alkaline medium following a 2-h extraction with a 0.01 M CaCl_2_ (Houba et al. 2000). The organic matter content was found to be 1.5% using an acidified solution of ferrous ammonium sulfate method after a digestion of the soil sample with an acidified dichromate (Bremner 2018). To prepare the soil sample for further analysis, it was diluted with sterile distilled water (SDW) and subjected to vortexing for 3 min. The diluted sample was then spread onto a potato dextrose agar (PDA) medium supplemented with 10 mg/l rifampicin and 200 mg/l ampicillin. The plates were incubated at room temperature in the laboratory for a period of 5–7 days. Fungal colonies present on the plate were subjected to repeated streaking onto a new plate until pure cultures were achieved.

Subsequently, a total of seven distinct fungal strains exhibiting different morphologies were individually assessed for their pathogenicity. The fungi were individually cultured on PDA for a period of 3–4 days at a temperature of 28°C, following which spores were harvested and preserved in SDW at 4°C before utilization. Seedlings of *CA* plants, possessing three to four leaves and cultivated in quartz sand, were then transferred to pots containing 2 kg of soil each. The soil utilized for the pot experiment was collected from the campus of Yancheng Teachers University. Each of the seven fungal spore suspensions was inoculated into five pots, with the final concentration of 10^9^ colony-forming units (CFU) per kg of soil, specifically around the root area. Concurrently, a control group of soil without inoculation was employed. The disease severity index of *CA* leaves was assessed on a scale ranging from 0 to 4, where 0 = no disease symptom, 1 = 0.1%–5%, 2 = 5.1%–20%, 3 = 20.1%–40%, and 4 = 40.1%–100% (the percentage of diseased leaf area). The disease severity value was determined utilizing the subsequent formula: Disease index (%) = [Σ (the number of diseased leaves × disease severity index)/(4 × the number of leaves evaluated)] ×100 (Lee et al. 2006). Subsequent to the pathogenicity analysis and symptom observation, the fungal pathogen exhibiting the highest disease index was chosen and subsequently reisolated from the plant roots. Subsequently, the reisolated strain was subjected to testing to ascertain its pathogenicity towards *CA*.

The genomic DNA of the chosen pathogenic fungus was extracted utilizing a Rapid Fungi Genomic DNA Isolation Kit (Sangon Biotech Co., Ltd., Shanghai, China) in accordance with the manufacturer’s instructions. The internal transcribed spacer gene (*ITS*) region of the ribosomal RNA, translation elongation factor 1-α gene (*TEF1*), and the 6–7 region of RNA polymerase II gene (*RPB2*) were amplified using the universal primers ITS1/ITS4 (forward primer: 5′-TCCGTAGGTGAACCTGCGG-3′, reverse primer: 5′-TCCTCC GCTTATTGATATGC-3′) (White et al. 1990), EF1-983F/EF1-1567R (forward primer: GCYCCYGGHCAYCGTGAYTTYAT, reverse primer: ACHGTRCCRATACCACCSATC) (Rehner and Buckley 2005), and RPB2-b6F/RPB2-b7.1R (forward primer: 5′-TGGGGYATGGTNTGYCCYGC-3′, reverse primer: 5′-CCCATRGCYTGYTTMCCCATDGC-3′) (Matheny 2005), respectively. The resulting PCR products were subsequently submitted to Sangon Biotech. Co., Ltd for sequencing, and the obtained sequences were deposited in the NCBI database with the accession numbers PP577661 (*ITS*), PP692189 (TEF1), and PP692190 (RPB2). Subsequently, phylogenetic trees were constructed using the DNA sequences through the employment of Mega X software and the neighbor-joining method (Kumar et al. 2018). The identification of the fungal strain responsible for the root rot disease in *CA* was conducted, resulting in the identification of *Fusarium solani* as the causative agent.

### Preparations of leaf, root extracts, and fungal pathogen inoculum

Fallen yellow leaves and mature roots were collected from the planting base and subjected to grinding and drying at a constant weight of 65°C. A total of 30 g of dried leaves or roots were then immersed in 1 l of SDW at a temperature of 24°C for a duration of 48 h, with periodic shaking every 12 h. Following the 48-h incubation period, the liquid was subjected to centrifugation at a speed of 10 000 r/min for a duration of 20 min, and the resulting supernatant was collected. The extracts were concentrated 3-fold over an 18-h period using a vacuum freeze dryer, resulting in a concentration of 90 g/l as determined by the oven-drying method at 105°C for 5–6 h. Thus, the water extracts of leave extracts (LE) and root extracts (RE) were obtained separately and stored at 4°C for future use. The extracts were divided into two portions, filtered through a 0.22-µm pore size membrane, and autoclaved. *Fusarium solani* D1 was cultured on PDA media at 28°C for a duration of 3 days. Subsequently, spores from the plates were collected using SDW and adjusted to a concentration of 10^8^ CFU/ml. The spores were also stored at 4°C for <1 week.

### Effects of LE, RE, and D1 on seed germination

A minimum of 360 seeds of *CA* (variety: Binwu No. 1) was subjected to surface sterilization using a 50% bleach solution for 15 min, followed by three washes with SDW. The surface sterilized seeds were then stored at 4°C overnight before utilization. Half the seeds were soaked in the spore suspension (10^8^ CFU/ml) for 20 min at room temperature, and then air dried. Meanwhile, the leftover seeds were soaked in SDW under the same conditions. These two portions were regarded as inoculated and uninoculated seeds, respectively. LE and RE were diluted to a concentration of 30 g/l to create stock solutions. Six treatments were established, including the control (consisting of uninoculated seeds receiving 2 ml of SDW every 2 days), D1 group (consisting of inoculated seeds receiving 2 ml of SDW every 2 days), LE group (consisting of uninoculated seeds receiving 2 ml of LE stock solution every 2 days), LE + D1 group (consisting of inoculated seeds receiving 2 ml of LE stock solution every 2 days), RE group (consisting of uninoculated seeds receiving 2 ml of RE stock solution every 2 days), and RE + D1 group (consisting of inoculated seeds receiving 2 ml of RE stock solution every 2 days). A total of 20 seeds were placed on a sterile filter paper in a Petri dish containing various additives as indicated above, and were subsequently subjected to germination at a temperature of 24°C. Each treatment and control were replicated three times in the experiment. On the 10th day, the germination rate of each Petri dish was determined. Additionally, in order to assess the heat resistance of the active substances in the extracts, autoclaved and filtered extracts were compared through the germination experiment.

To validate the findings of the aforementioned experiment, a supplementary pot experiment was conducted. Each treatment included six replicates, with 15 seeds sown in a pot with 2 kg of campus soil. The treatments remained consistent with those previously enumerated. To incorporate the extracts, 667 ml of both LE and RE (90 g/l) were uniformly added to 2 kg of soil, resulting in a final concentration of 30 g/kg of extracts in the soil. In the D1-containing treatments, the soil surrounding the seeds was supplemented with D1 spore suspensions at a final concentration of 10^9^ CFU/kg. On the 15th day, the germination rates were computed by considering the number of seedling emergence.

### Effects of LE, RE, and D1 on photosynthetic pigment contents

In light of the susceptibility of certain plant indicators to environmental stress, the levels of Chlorophyll Chl *a*, Chl *b*, carotenoid (Car), and phenylalanine ammonia lyase (PAL) were assessed over a period of time. A subsequent pot experiment was conducted, following the aforementioned protocol. The only deviation was the prearrangement of seedlings at the third true leaf stage, which were subsequently transplanted into individual pots (one seedling per pot) for this particular experiment. A minimum of 12 pots were prepared for each treatment. The methodology for the introduction of extracts and D1 remained consistent with the previously outlined procedure. At three time points, specifically day 0 (within 30 min after additions), day 15, and day 50, samples were collected from three randomly selected pots for each treatment. Additionally, soil samples were obtained from the same three pots. Soil sampling was conducted using a soil corer with an inner diameter of 3 cm, at a depth of 10 cm around the roots of each plant. Following the removal of root residues, the soil samples were divided into two portions. One portion was stored at 4°C for enzymatic activity determination within 1 week, while the other portion was stored at −80°C for molecular analysis.

For the assays of photosynthetic pigment contents, a updated procedure with new equations were adopted (Chazaux et al. 2022). The equations for quantifying chlorophyll a (Chl *a*), chlorophyll b (Chl *b*), and carotenoid (Car) are: Chl *a* = 12.18 × *A*_663.6–_2.36 × *A*_646.6_; Chl *b* = 20.19 × *A*_646.6–_4.59 × *A*_663.6_; and Car = (1000 × *A*_470–_2.05 × Chl *a* –114.8 × Chl *b*)/245, where A = absorbance in 1.00-cm cuvettes.

### Effects of LE, RE, and D1 on plant defense-related enzymes

In the second pot experiment, the determination of plant defense-related enzymes, specifically superoxide dismutase (SOD) and peroxidase (POD), was conducted (Datt 1999, Huang et al. 2010, Kong et al. 2014, Zameer et al. 2022). The preparation of crude extracts of these antioxidant enzymes involved homogenization of frozen leaves in a buffer medium. Specifically, a 10-g sample was homogenized in a 100-mM sodium phosphate buffer (pH 7.0) containing 1 mM ascorbic acid and 0.5% (w/v) polyvinylpyrrolidone for a duration of 5 min at a temperature of 4°C. The resulting homogenate was then filtered through three layers of gauze towel, and the filtrate was subsequently subjected to centrifugation at 5000 × *g* for a duration of 15 min, after which the supernatants were collected. The activity of PAL was assessed through the quantification of the absorbance of transcinnamic acid at a wavelength of 290 nm, as modified by González-Mendoza et al. (2018). A single unit (U) of this enzymatic activity was defined as the quantity that induces a 0.01 increase in absorbance per hour. The determination of SOD involved measuring the inhibition of nitro-blue tetrazolium (NBT) photoreduction by the SOD enzyme (Kumar et al. 2012). One unit (U) of SOD activity was defined as the quantity of enzyme that causes a 50% inhibition of the photochemical reduction of NBT. The determination of POD activity involved the measurement of the increase in absorption at 420 nm spectrophotometrically, which resulted from the oxidation of 4-methylcatechol by H_2_O_2_. The enzymatic activity was quantified as one U, defined as a 0.001 change in absorbance per minute (Onsa et al. 2004).

### Effects of LE, RE, and D1 on soil enzymes

Invertase activity was assessed using the 3,5-dinitrosalicylic acid colorimetric method and expressed as the amount of released reducing sugars derived from a 10% sucrose solution per gram of soil per 24 h at 37°C (Frankeberger and Johanson 1983). Urease activity was determined through the colorimetric measurement of ammonium produced from a 0.72-M urea solution and expressed as the amount of NH_3_-N in 1 g of soil after 24 h (Kandeler and Gerber 1988). Due to the alkaline nature of the soil samples, only alkaline phosphatase activity was detected. This was achieved by utilizing 1 mM *p*-nitrophenyl phosphate (*p*NPP) as a chromogenic substrate, and the activity was quantified as mg *p*-nitrophenol (*p*NP) per gram of soil per 24 h (Li et al. 2016a).

### Quantitative polymerase chain reaction analysis of D1

To extract the total microbial DNA from ∼0.5 g of soil, a PowerSoil^®^ DNA Isolation Kit (MO BIO Laboratories Inc., CA, USA) was employed, and the extracted DNA was assessed using agarose gel electrophoresis. For the quantification of the abundance of strain D1, a primer pair FSGq1 (5′-GGCTGAACTGGCAACTTGGA-3′) and FSGq2 (5′-CAAAGCTTCATTCAATCCTAATACAATC-3′) (Li et al. 2008), together with a specific probe (5′-6FAM-TCTTCTAGGATGGGCTGGT-MGBNFQ-3′) (Li and Hartman 2003) targeting the minor groove-binding region were utilized in quantitative polymerase chain reaction (qPCR) analysis. A volume of 1 µl of DNA was added to a 25-µl qPCR reaction mixture using the OmniMix HS Beads (Cepheid, Sunnyvale, CA). Each DNA sample was tested three times by qPCR. The reaction conditions were as follows: 95°C for 120 s, then 45 cycles of 95°C for 120 s and 60°C for 30 s. A standard curve of strain D1 DNA was prepared in triplicate using the probe in qPCR. Specifically, a 10-ng/µl solution of the DNA of strain D1 was diluted 10-fold serially to 10^−6^ ng/µl. It was calculated directly from the concentration of extracting plasmid carrying target genes from soil samples according to the standard method. A volume of 1 µl DNA from each dilution was added to the OmniMix HS master mix and each dilution was tested three times. The DNA concentrations of strain D1 and resulting threshold cycle (*C*_t_) values were used to construct a standard curve.

### Detections of phenolic acids in soil and plant tissues

On 10 September 2022, soil samples (0–20 cm) from a planting base (120.29523 E, 34.20 406 N) were collected at different durations of *CA*: 0 years (0 a), 2 years (2 a), and 3 years (3 a). The quantification of phenolic compounds was conducted using high-performance liquid chromatography (HPLC). Leaf or root samples weighing 2.0 g were placed into a 50-ml graduated plastic test tube and homogenized in a 7-ml methanol solution (adjusted to pH = 2 with 1 mol/l HCl) using an ultrasonic homogenizer (Hielscher-UP200 Ht, Germany) at a temperature of 45°C for a duration of 40 min. The mixture was then cooled to room temperature and diluted to a final volume of 10 ml with SDW. Following centrifugation at a speed of 10 000 r/min for 20 min, 1 ml of the resulting supernatant was filtered using a 0.22-µm pore size filter. (Mattila and Kumpulainen 2002). The obtained results were subsequently adjusted based on the weight of the dried samples.

The HPLC analysis was conducted using a SunFire^TM^ C18 column (4.6 mm × 250 mm, 5 µm) on an Agilent 1200 instrument (Agilent Technologies, Santa Clara, CA, USA). The analysis employed an injection volume of 10 µl, a flow rate of 0.6 ml/min, a column oven temperature of 30°C, and a UV detection wavelength of 280 nm. The elution mobile phases consisted of methanol (phase A) and a 1% aqueous acetic acid solution (pH 2.5) (phase B), with retention times ranging from 0 to 30 min. The ratio of mobile phase A to B was established as 1:3. To mitigate the influence of interfering components and ensure result stability and repeatability, a 10-min delay was implemented at the conclusion of each cycle. Based on the findings of prior research (Tian et al. 2015, Bai et al. 2019), a subset of seven phenolic acids were selected as standard samples for analysis. These standard samples, obtained from Sigma, consisted of coumaric acid, *p*-coumaric acid, caffeic acid, *p*-hydroxybenzoic acid, vanillin, ferulic acid, and syringic acid. The quantification of phenolic acids was performed utilizing the external standard method, while the identification of samples was accomplished by assessing the retention time and peak area of the analytical standards (Bai et al. 2019).

### Effects of phenolic acids and extracts on the mycelium growth of strain D1

The present study investigated the effects of four identified phenolic acids (*p*-hydroxybenzoic acid, caffeic acid, *p*-coumaric acid, and ferulic acid) on the growth of *F. solani* D1 mycelium. To accomplish this, mycelia exhibiting equivalent growth from the periphery of the colony were extracted using a 5-mm diameter hole punch and subsequently introduced into PDA media supplemented with varying concentrations (0, 2, and 6 mg/l) of the aforementioned phenolic acids. Subsequently, the diameters of the colonies were measured after 2, 3, and 4 days of cultivation at a temperature of 28°C.

### Effects of LE and RE on the growth of strain D1

LE or RE were separately added to 50 ml of PDA liquid media at four different final concentrations (0, 10, 20, and 30 g/l). The media were then inoculated with strain D1 at a final concentration of 10^6^ CFU/ml. The triangular flasks containing the cultures were incubated at 28°C and 180 r/m. Culture mixtures from each treatment were collected at intervals of 4–12 h using a pipette with 2 mm truncated tips, followed by centrifugation at 8000 r/min for 15 min. The resulting precipitates were washed with SDW and subsequently dried at 80°C overnight until a constant weight was achieved. The dry weights of the hyphae were then measured.

The method used to investigate the effects of LE or RE on the mycelium growth of strain D1 followed the same procedure as previously described. Colony diameter was measured at 84 h for each plate. Additionally, spore numbers on each plate were counted using a Sysmex-5000i automated hematology counter after spore washing with SDW. Furthermore, spore germination rates were determined using a hemocytometer after 24 h of culture in PDB media containing filtrated LE or RE at final concentrations of 10, 20, and 30 g/l, following inoculation with the same number of spores of strain D1.

### Effects of LE and RE on the activities of pathogenesis-related enzymes

To investigate the impact of LE and RE on enzymes implicated in pathogenesis, the activities of four invasion-related enzymes (pectinase, cellulase, β-glucosidase, and α-amylase) were assessed and compared across various treatments (Zhang et al. 2006, Hasan et al. 2013, Bethke et al. 2016, Huang et al. 2021). Utilizing the growth curve determination system previously described, a volume of 2 ml of the culture solution was transferred via pipette into a sterile tube and subsequently centrifuged at 8000 r/min for a duration of 20 min. The resulting supernatant was then utilized as the crude enzyme for subsequent analysis. Pectinase activity was measured by quantifying the reducing sugars generated through enzymatic hydrolysis of pectin using the DNS method (Miller 1959). Pectin was utilized as the substrate for determining pectinase activity. The reaction mixture, consisting of an equal amount of substrate (2%) prepared in citrate buffer (0.05 mol/l, pH 4.4), and appropriately diluted enzyme, was incubated at a temperature of 50°C for a duration of 30 min in a water bath. The quantification of reducing sugars released was conducted using the DNS method, with galacturonic acid serving as the standard. The enzymatic activity of pectinase was determined as the quantity of enzyme necessary to liberate 1 µmol equivalent of galacturonic acid per minute under the specified assay conditions. The cellulase activity was conducted using a citrate buffer (0.05 mol/l, pH 4.8). The enzyme was appropriately diluted and incubated at 50°C for 60 min in a water bath. The activity was measured using the DNS method, with a 50-mg Whatman No. 1 filter paper strip (1.0 cm × 6.0 cm) serving as the substrate. The cellulase enzymatic activity was defined as the quantity of enzyme necessary to reduce sugars equivalent to 1 µg of glucose per ml per minute (Ghose 1987). The β-glucosidase activity was carried out in an acetic acid buffer (50 mmol/l, pH 5.0). The enzyme was suitably diluted and incubated at 60°C for 10 min in a water bath. The activity was determined by measuring the hydrolysis of *p*-nitrophenyl β-d-glucopyranoside. The activity of β-glucosidase was quantified as the quantity of enzyme that liberates 1 µmol *p*-nitrophenol per minute, representing 1 U of enzymatic activity (Cai et al. 1999). Meanwhile, the activity of α-Amylase was assessed by measuring the conversion of starch into glucose using the DNS method. One unit of α-Amylase activity was defined as the amount of reducing sugar (calculated as maltose) released from starch, equivalent to 1 mg, per ml per minute under the specified experimental conditions (Lorentz 1979).

### Scanning electron microscopy observation of the morphologies of strain D1 following different treatments

Strain D1 seed cells were evenly inoculated onto PDA plates containing varying concentrations of LE and RE (0 and 30 mg/l) and incubated at a temperature of 28°C for a duration of 84 h. Hyphae from each plate were carefully selected and placed onto a coverslip, which was subsequently fixed in a solution of 2.5% glutaraldehyde in 0.01 mol/l PBS for a period of 1 h at room temperature. The coverslip was then sequentially immersed in ethanol solutions of increasing concentrations (30%, 50%, 70%, 80%, and 90%) for 10 min each, followed by dehydration with anhydrous ethanol for 10 min, repeated two to three times. Subsequently, the samples were placed onto the objective table for observation after being coated with gold, with a working distance of 6.6 mm and a voltage of 3.0 kV.

### Microbial community analysis

Microbial DNA were extracted from four pot soil samples (control, D1, LE, and LE+D1) at different sampling points (0, 15, and 50 d) using the E.Z.N.A.^®^ Soil DNA Kit. 16S rRNA and ITS genes were amplified with the primer sets: 515f (5′-GTGCCAGCMGCCGCGGTAA-3′) and 907r (5′-CCGTCAATTCMTTTRAGTTT-3′) (Klindworth et al. 2013), ITS3-F: (5′-GCATCGATGAAGAACGCAGC-3′) and ITS4-R: (5′-TCCTCCGCTTATTGATATGC-3′) (McKay et al. 1999), respectively, and then added with index codes. A 25 µl of reaction mixture contained 5 µl 5x reaction buffer, 5 µl 5x GC buffer, 2 µl dNTP (2.5 mM), 1 µl of each primer (10 µM), 2 µl template DNA, 0.25 µl Q5^®^ High-Fidelity DNA Polymerase (New England Biolabs, Ipswich, MA, USA), and 8.75 µl ddH_2_O. The temperature cycle comprised of an initial step at 98°C for 1 min, followed by 30 cycles of 98°C for 15 s, 55°C for 30 s, 72°C for 30 s, and a final extension at 72°C for 10 min. The bacterial or fungal communities in each sample were assessed using the Illumina MiSeq System (Biozeron Biotechnology Co., Ltd, Shanghai, China). The gene sequences were selected based on a 97% identity threshold after consolidating the original data and applying filtering and quality assessment using QIIME (v1.8.0, http://qiime.org/). The Ribosomal Database Project (RDP) (http://rdp.cme.msu.edu/) was utilized for species annotation analysis. Filtered reads were clustered into operational taxonomic units (OTUs) assuming 97% similarity cutoff using uclust (Edgar 2010), and the OTUs with average abundances below 0.0001% per sample were excluded (Bokulich et al. 2013). A total of 509 080 and 398 139 high-quality sequencing reads were acquired for subsequent analysis. Prior to assessing soil microbial diversity, the microbial community data underwent resampling to ensure a consistent minimum number of sequences per sample, specifically 6803 for bacteria and 10 047 for fungi.

### Data analysis

The raw data were imported into SPSS Statistics for Windows version 18.0 (WinWrap Basic, Polar Engineering and Consulting) in order to calculate means and standard errors (SE). A phylogenetic tree was constructed using DNA sequences through the utilization of Mega X software’s neighbor-joining method. Principal component analysis (PCA) was conducted in R v.3.2.1 with the ggplot2 package (Wickham 2016), which relies on the average covariance matrix. A two-way analysis of variance (ANOVA) program in SPSS was utilized to examine the impact of extract types [no extract, low extract (LE), or high extract (RE)] and pathogen presence (with or without) on the plant growth indices. Similarly, a two-way ANOVA was conducted to assess the effects of extract types (LE and RE) and varying amounts (0, 10, 20, 30 g/l) on the growth and activity of pathogenesis-related enzymes of strain D1. Prior to this analysis, normality was tested using the Shapiro–Wilk normality test, and a *P-*value of < .05 was considered significant. Subsequently, Bonferroni’s *post hoc* tests were conducted. In each plot, different asterisk symbols (*, **, and ***) indicate significant differences between samples at significance levels of .05, .01, and .001. Significant differences were observed at one sampling time for certain plots, as indicated by distinct letters (Duncan’s multiple range test, *P* ≤ .05). Permutational multivariate analysis of variance (PERMANOVA) was conducted using the command “adonis” in the “vegan” package of R to assess the effects of the different additives on the changes in the bacterial or fungal community. The line and column charts utilized in this study were generated using Sigma Plot for Windows Version 10.0 (Systat Sofware, San Jose, CA, USA). The Shannon index was computed using Mothur1 (Schloss et al. 2011).

## Results

### Isolation and identification of fungal pathogen causing root rot disease

**Figure 1.**
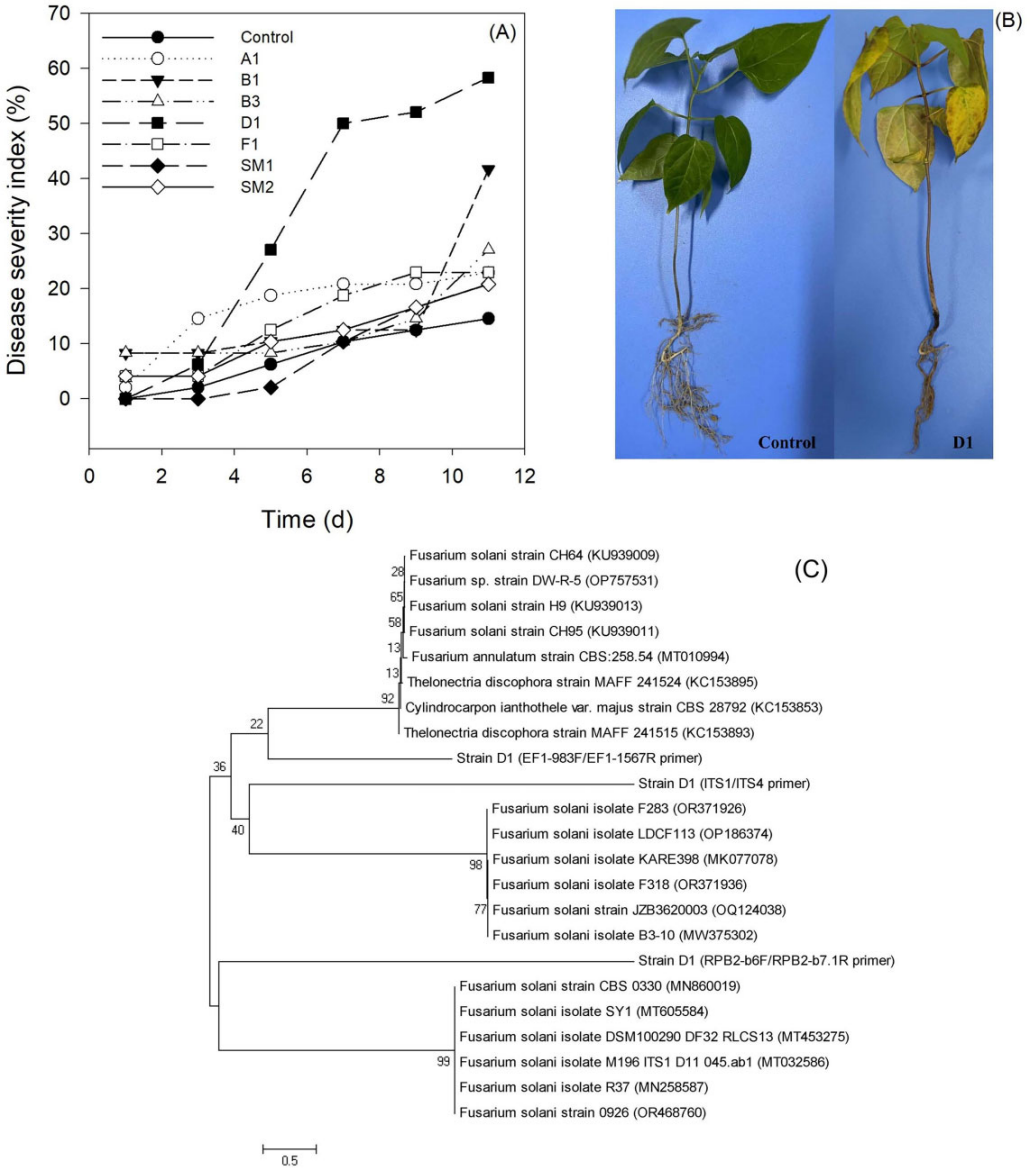
Isolation and identification of a fungal pathogen causing root rot disease. (A) Disease severity index with time after inoculation with different fungal strains at the final concentration of 10^9^ CFU/kg soil. (B) Symptom of root rot disease caused by strain D1 on day 11. (C) Phylogenetic tree based on the sequences of ITS region of strain D1 and its relatives by using neighbor-joining method.

Of the seven potential pathogenic fungi associated with root rot disease, strain D1 was selected for its notable pathogenicity towards *CA* (Fig. 1A). Subsequently, strain D1 was verified in accordance with Koch’s postulates, and the symptom induced by D1 was found to be consistent with root rot disease (Fig. 1B), characterized by the presence of circular or irregular light brown lesions that develop into dark black lesions on subterranean roots and stems, resulting in stunted growth and eventual mortality. Subsequent identification of strain D1 as a member of *F. solani* was achieved through sequences of the three representative genes (Fig. 1C) and the observation of falciform spores (Fig. 5E).

### Effects of LE, RE, and D1 on seed germination

**Figure 2.**
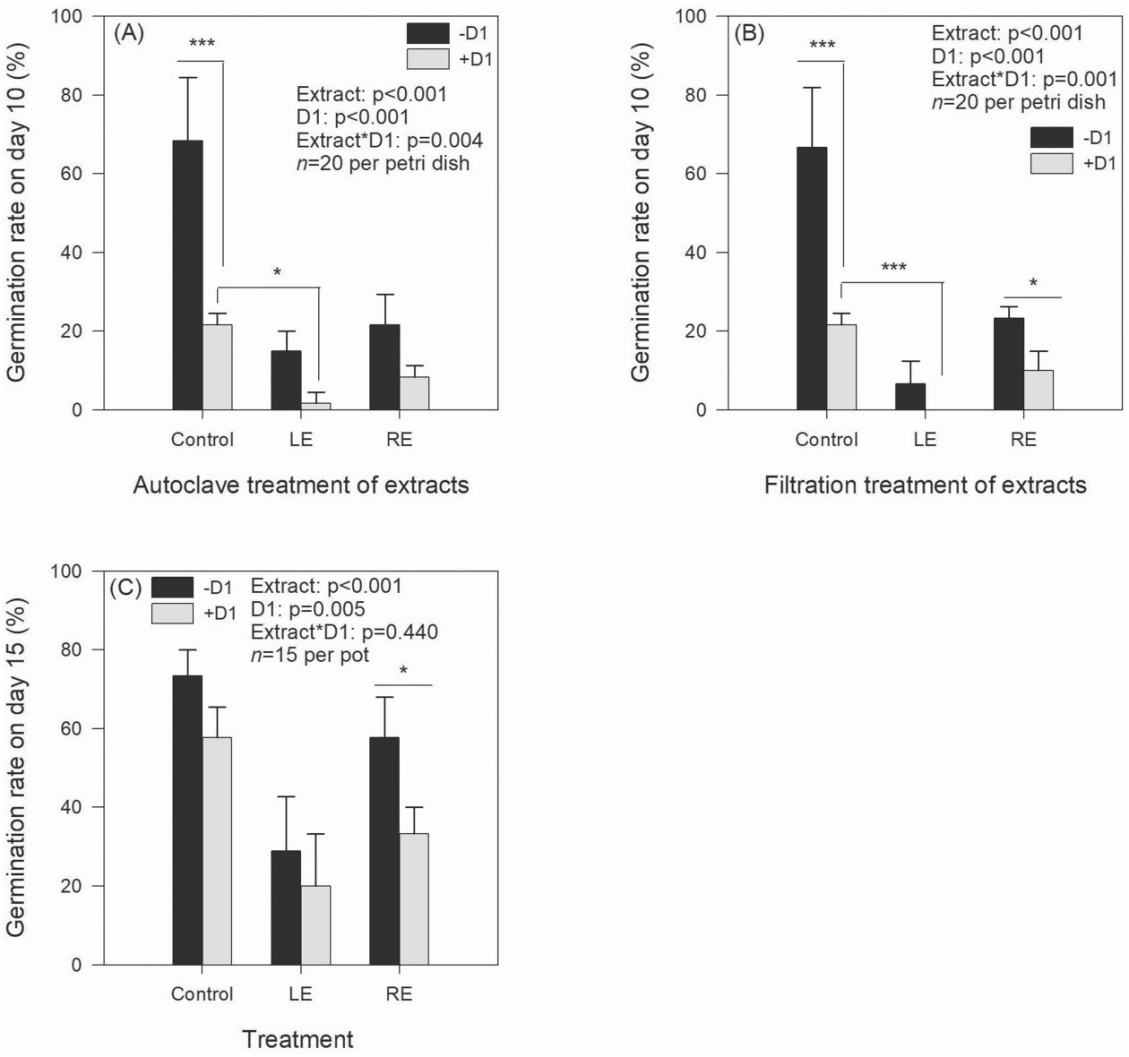
Effects of LE and RE on seed germination using petri dishes (A, B) and pots (C) experiments. A two-way ANOVA was performed to test the effects of extract kinds (LE or RE) and D1 (with or without) on the indices, followed by Bonferroni’s *post hoc* tests. “−D1” and “+D1” in panels (A) and (B) indicated treatments without and with the spores of strain D1 at the final concentration of 10^6^ CFU/ml. Different numbers of asterisk (*, **, and ***) represent significant differences between samples at *P*-values of .05, .01, and .001.

Strain D1 exhibited a significant reduction in the germination rate of seeds, with a decrease of 68.29% compared to the control group without D1 addition (*P* < .001) (Fig. 2A). Autoclave treatment of LE and RE also resulted in an inhibition of seed germination rates, with reductions of 78.05% and 68.29%, respectively. Filtration treatments of LE and RE yielded similar effects to the autoclave treatments (Fig. 2B). Both LE and RE demonstrated a synergistic effect with strain D1 on seed germination. The overall trend in the influence of LE and RE on seed germination in soil closely mirrored the results obtained from the filter paper assay (Fig. 2C). Nevertheless, the adverse effects of D1 were not statistically significant (*P* > .05) when compared to noninoculation treatments, with the exception of the RE treatment.

### Effects of LE, RE, and D1 on photosynthetic pigments in leaves of CA

**Figure 3.**
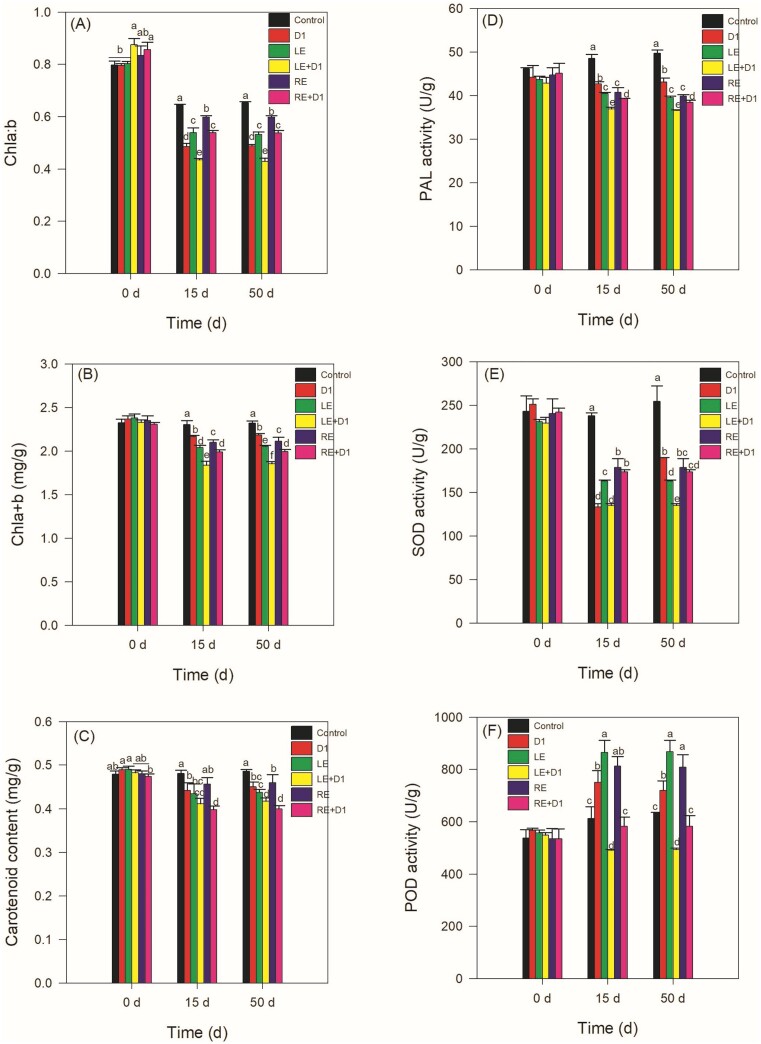
Plant stress responses to LE, RE, and/or strain D1. The changes in the contents of photosynthetic pigments of Chl *a:b* (A), Chl *a*+*b* (B), and carotenoid content (C) in *CA* plants following the addition of plant extracts and/or strain D1. The activities of defensive enzymes following the additions of plant extracts and/or strain D1(D–F). Legends shown in panel (A) apply equally to panels (B–F). Different letters at one sampling time point indicates significant differences among treatments (Duncan’s multiple range test, *P* ≤ .05).

On day 0 (the time period immediately following the introduction of additions within 30 min), the Chl *a:b*, Chl *a+b*, and carotenoid levels in the D1 and LE treatments did not exhibit any significant differences when compared to the control group (*P* > .05, Figs 3A–C). However, the Chl *a:b* ratio of the LE + D1 treatment was significantly higher than that of the LE treatment (*P* < .05, Fig. 3A), which was not observed in the RE treatments. Additionally, the three indices of the LE and RE treatments, with the exception of carotenoid content in the RE treatment on day 15, were found to be significantly lower than those of the control group on days 15 and 50 (*P* < .05). The presence of strain D1 further intensified this situation.

### Effects of LE, RE, and D1 on the activities of defense-related enzymes in CA

**Figure 4.**
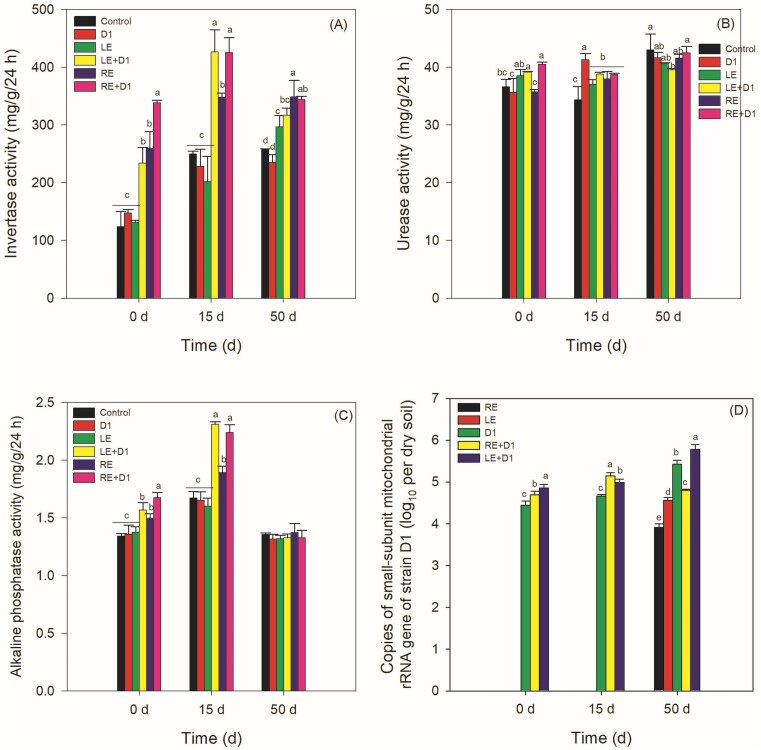
Effects of LE and RE on soil enzymes activities (A–C) and abundances of strain D1 using qPCR method (D). Legends shown in panel (A) apply equally to panels (B) and (C). Different letters at one sampling time point indicates significant differences among treatments (Duncan’s multiple range test, *P* ≤ .05).

There were no statistically significant differences observed among the treatments on day 0 (*P* > .05, Figs 3D–F). The activities of PAL and SOD in the control group were found to be the highest on days 15 and 50, as shown in Figs 4(D) and (E). Both LE and RE treatments resulted in a significant decrease in the activities of these two enzymes (*P* < .05), and when combined with strain D1, the inhibitory effects were further exacerbated. Strain D1, LE, and RE treatments led to a significant increase in POD activities on days 15 and 50 compared to the control group (*P* < .05, Fig. 3F). However, the combination of LE or RE with D1 also resulted in a significant decrease in the values of POD (*P* < .05).

### Effects of LE, RE, and D1 on soil enzymatic activities

The additions of LE + D1, RE, and RE + D1 led to notable rises in invertase activities on day 0 (*P* < .05, Fig. 4A). The invertase activities of RE + D1 exhibited a statistically significant increase compared to RE on day 0 and 15 (*P* < .05), but not on day 50. Furthermore, the enzymatic activity experienced a significant enhancement by LE up to day 50 (*P* < .05), with a slight additional increment when combined with the mixture of LE and D1. The treatments of LE, LE + D1, and RE + D1 resulted in significant increases in urease activities compared to the control group on day 0 (*P* < .05, Fig. 4B). By day 15, all treatments showed significant increases compared to the control group (*P* < .05), with D1 demonstrating the most pronounced effect. By day 50, the inhibitory effect of LE + D1 on urease activity was significant (*P* < .05), whereas on days 0 and 15, it showed an increase compared to the control group. A similar trend was observed for alkaline phosphatase activity compared to invertase activity, except that no significant differences were observed among treatments on day 50 (*P* > .05, Fig. 4C).

### Effects of LE and RE on the abundances of strain D1 in soil

The LE + D1 treatment exhibited the greatest abundance of strain D1 on day 0, followed by the RE + D1 and D1 treatments (Fig. 4D). Strain D1 was not detected in the LE and RE treatments on days 0 and 15, but appeared on day 50 and reached varying abundances. The abundances of D1 in the LE + D1 treatments increased over time and reached their peak on day 15, surpassing the abundances observed in the other treatments.

### Effects of LE and RE on the growth of D1

**Figure 5.**
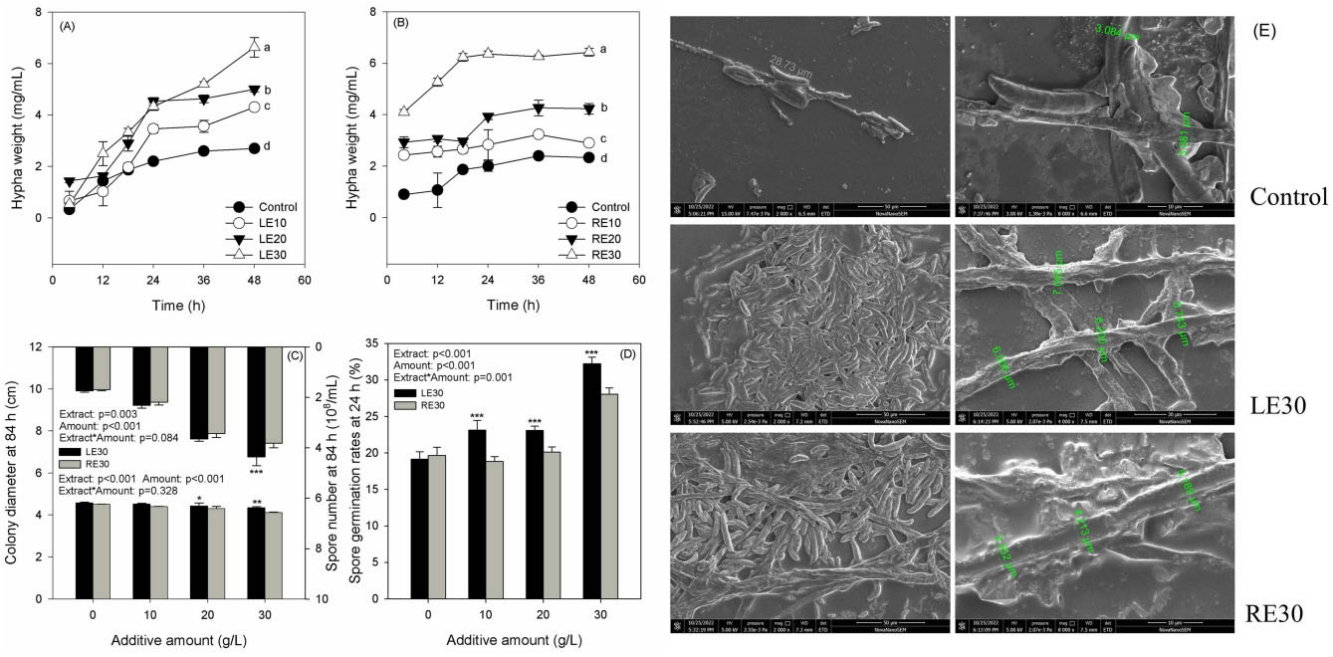
Effects of LE and RE on the growth of strain D1. Hypha weights in responses to LE (A) and RE (B), and the different letters on the plots indicates significant differences among treatments at 48 h (Duncan’s multiple range test, *P* ≤ .05). Effects of LE and RE on colonies diameters and spore numbers at 84 h (C), and spore germination rates at 24 h (D). (E) Morphologies of strain D1 following LE30 and RE30 treatments using SEM, and the pictures exhibiting the spores and mycelium of strain D1 were magnified by 2000 times and 8000 times, respectively. Different numbers of asterisk (*, **, and ***) represent significant differences between samples at *P*-values of .05, .01, and .001.

Both LE and RE demonstrated a concentration-dependent ability to enhance the hypha weights of strain D1, with RE exhibiting a more rapid effect compared to LE (Figs 5A and B). While LE or RE slightly inhibited colony diameters, the extracts significantly promoted spore numbers and the percentage of spore germination (*P* < .01), particularly LE at a concentration of 30 g/l (Figs 5C and D). Through scanning electron microscopy (SEM) observation, it was evident that the extracts, particularly LE30, significantly enhanced the spore numbers and hypha diameters. Specifically, the hypha diameter experienced an ∼50% increase (Fig. 5E).

### Effects of LE and RE on activities of pathogenesis-related enzymes in D1

**Figure 6.**
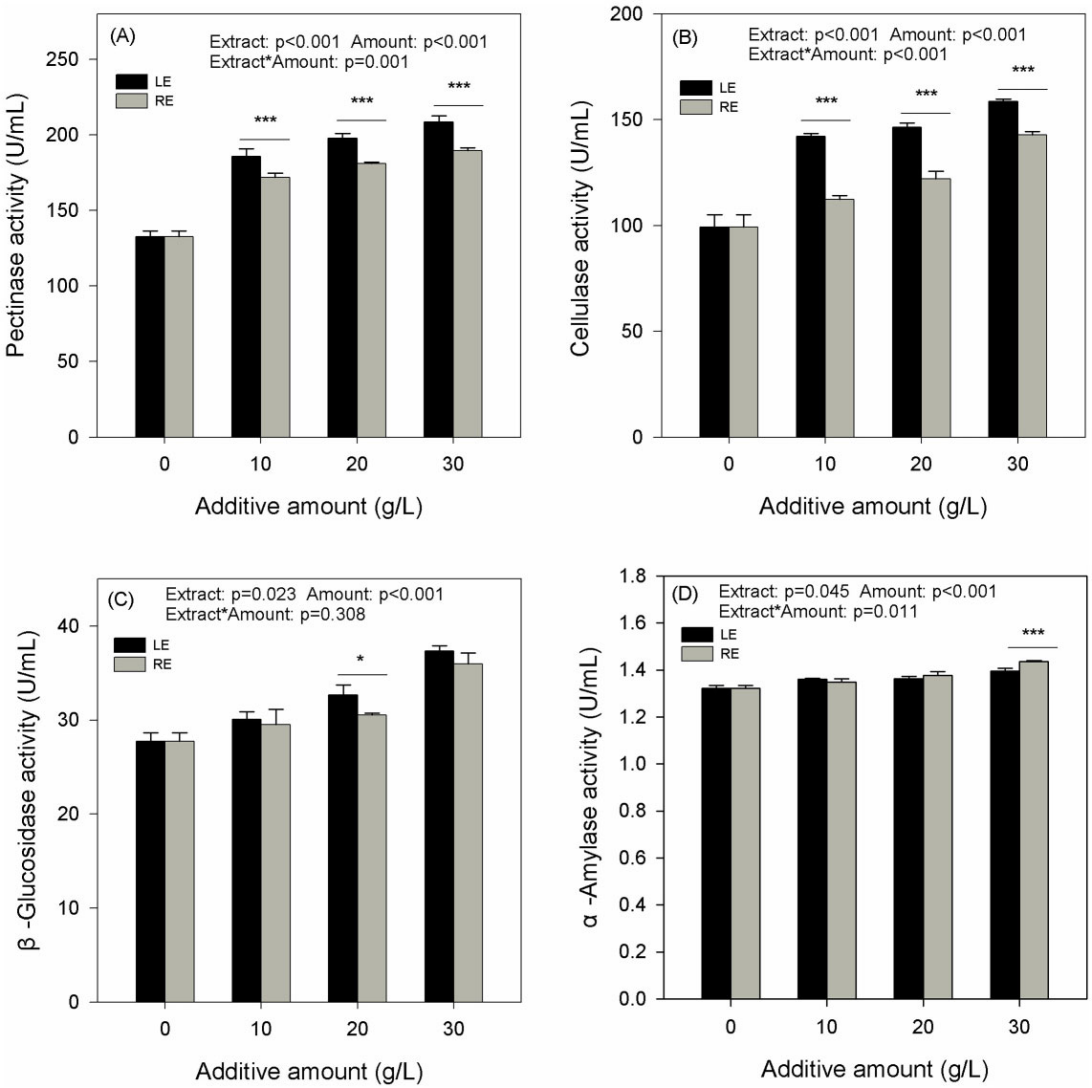
Effects of LE and RE on the pathogenesis-related enzymes activities of strain D1. Effects of different additives on strain D1 pectinase activity (A), β-glucosidase activity (B), cellulase activity, (C) and α-amylase activity (D). Different numbers of asterisk (*, **, and ***) represent significant differences between samples at *P*-values of .05, .01, and .001.

Both LE and RE demonstrated a concentration-dependent increase in the activities of pectinase and cellulase in D1. Notably, LE exhibited a greater promoting effect on D1 compared to RE (Figs 6A and B). Additionally, both LE and RE were found to promote the activities of β-glucosidase and α-amylase. However, LE did not consistently exhibit a significantly higher effect than RE, except at the concentration of 20 g/l for β-glucosidase (Figs 6C and D).

### Contents of phenolic acids in soils and extracts

**Figure 7.**
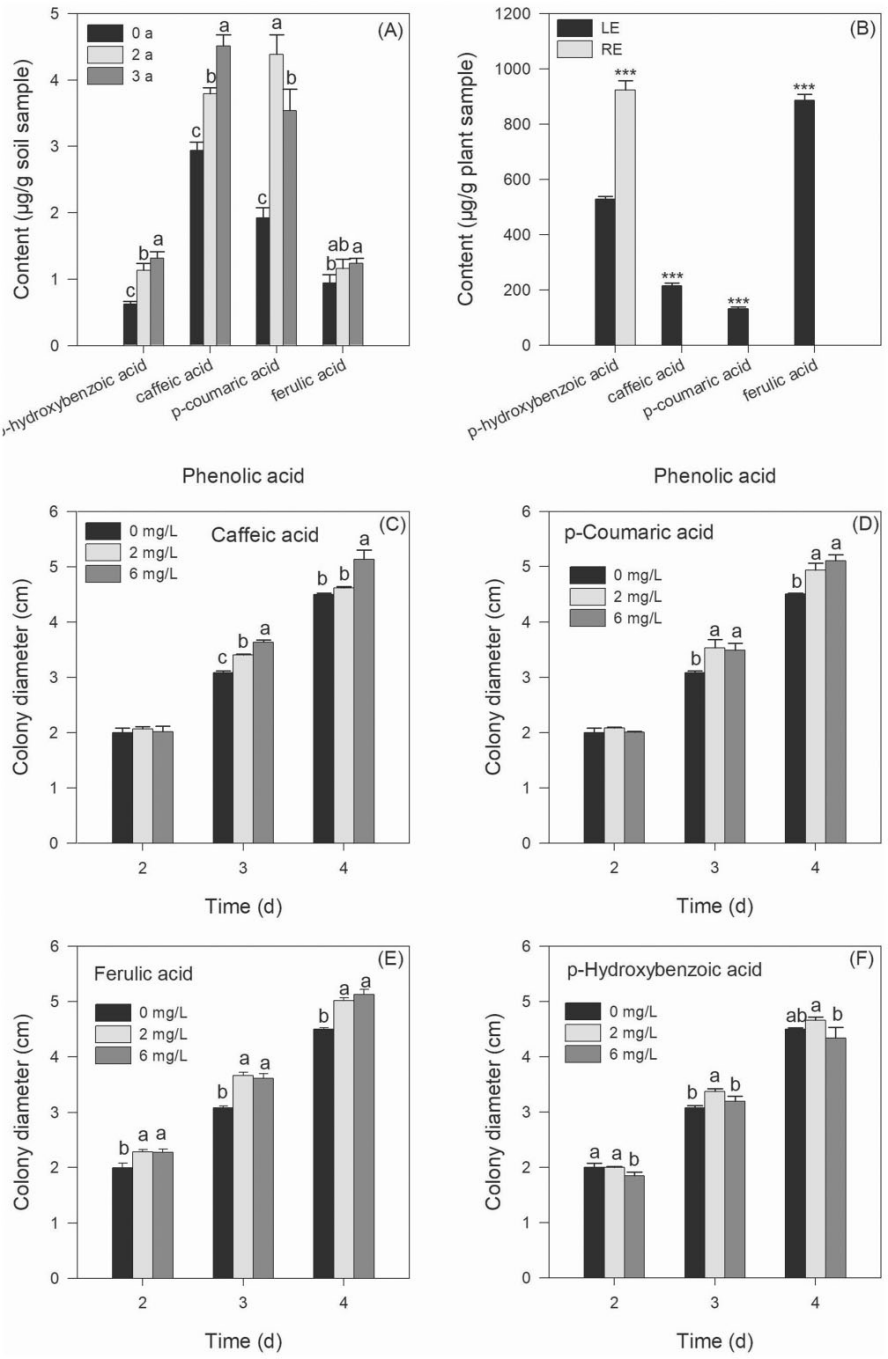
Contents of the main phenolic acids in soils with different *CA* cropping histories (A) and plant tissues (B), and their effects on the growth of stain D1 (C–F). In panel (A), 0 a, 2 a, and 3 a indicated that soils have been planted with *CA* for 0 year, 2 years, and 3 years, respectively. Different letters shown in panel (A) indicate significant differences in one certain phenolic acid among different cropping histories (Duncan’s multiple range test, *P* ≤ .05). Different numbers of asterisk (*, **, and ***) shown in panel (B) represent significant differences between samples at *P*-values of .05, .01, and .001. Different letters shown in panels (C–F) at one sampling time indicate significant differences among treatments (Duncan’s multiple range test, *P* ≤ .05).

In order to ascertain the resemblance of phenolic acids to previous findings, specifically in terms of heat resistance, year-on-year increment, and higher prevalence in LE compared to RE, we conducted a comparative analysis of phenolic acid contents in soils with diverse cropping histories of CA, as well as in LE and RE. The results revealed an increase in *p*-hydroxybenzoic acid and caffeic acid over time, while *p*-coumaric acid and ferulic acid exhibited greater abundance in soils subjected to continuous cropping in comparison to the control soil (Fig. 7A). Caffeic acid, *p*-coumaric acid, and ferulic acid were found to be abundant in LE and absent in RE, whereas *p*-hydroxybenzoic acid exhibited higher levels in RE compared to LE (Fig. 7B). The impact of these four phenolic acids on the growth of strain D1 was assessed using the index of colony diameters. Caffeic acid, *p*-coumaric acid, and ferulic acid were observed to stimulate the growth of D1 (Figs 7C–F).

### Profiles of the microbial communities following LE and D1 additions

**Figure 8.**
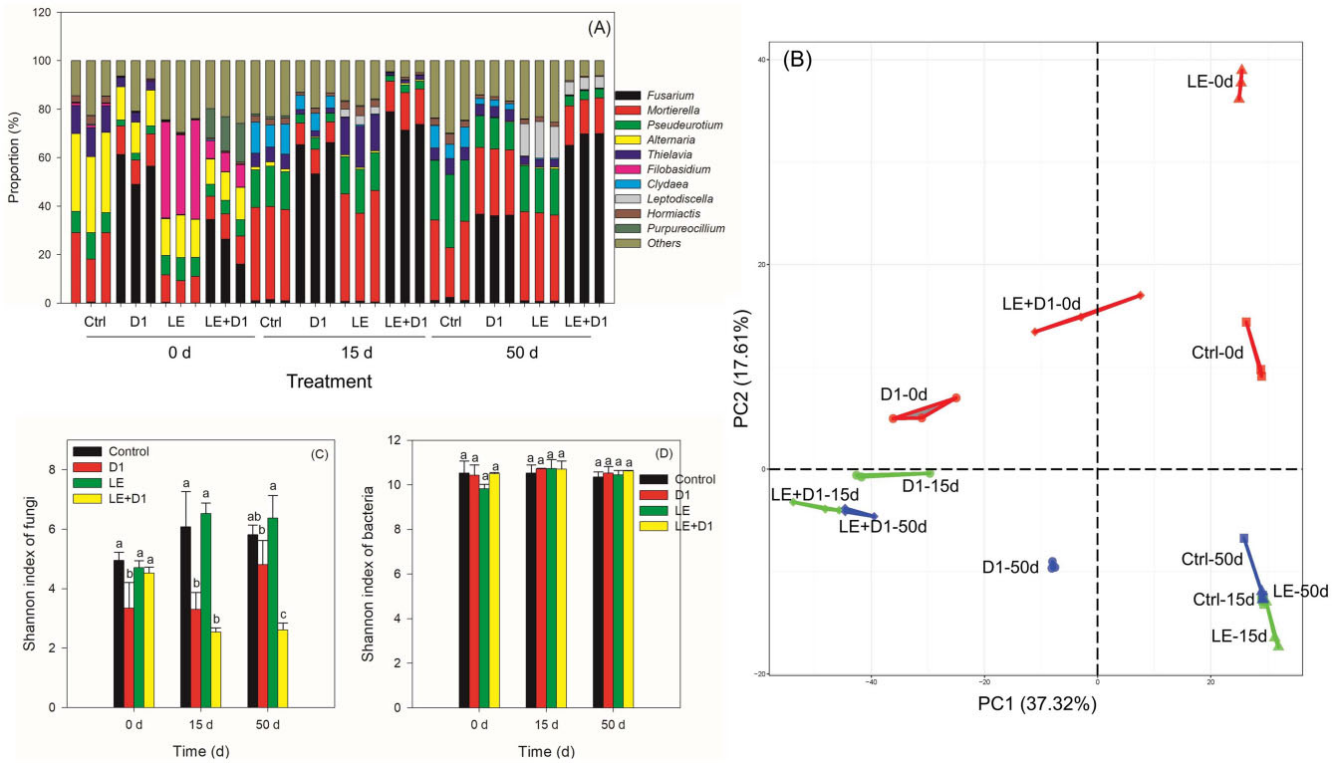
Effects of LE and/or strain D1 on the fungal community with time (A), the changes in fungal composition among different treatments during 50 days using PCA (B), and Shannon indices of fungi (C) and bacteria (D). Different letters shown in panels (C) and (D) at one sampling time indicate significant differences among treatments (Duncan’s multiple range test, *P* ≤ .05).

The control group had *Mortierella* and *Alternaria* as dominant fungi on day 0, shifting to *Mortierella* and *Pseudeurotium* on days 15 and 50 (Fig. 8A). *Filobasidium* decreased in the LE treatment, while *Fusarium* increased in the LE + D1 treatment on days 15 and 50. The top 30 genera in the treatments are shown in Fig. S3, with 20 belonging to the *Ascomycota* phylum. The Bray–Curtis dissimilarity-based PERMANOVA analysis revealed statistically significant impacts on the fungal community subsequent to the addition of LE or D1 (*P* < .0001, *R*^2^ = 0.936). Overall, the fungal communities on day 0 were distinct from those on days 15 and 50 (Fig. 8B). The introduction of LE resulted in a gradual return to control conditions, as evidenced by the clustering of Ctrl-15, Ctrl-50, LE15, and LE50 in the bottom right corner. The addition of D1 had a significant impact on the fungal community dynamics over time. Interestingly, the LE + D1 treatments on days 15 and 50 exhibited similar patterns of convergence. The introduction of LE or D1 induced alterations in fungal diversity, with LE + D1 consistently leading to a reduction in fungal diversity (Fig. 8C). Solely the D1 treatment exhibited a significantly lower fungal diversity than the other treatments on day 0. On day 15, both the D1 and LE + D1 treatments displayed significantly lower diversities compared to the control and LE treatment. On the 15th day, the D1 treatment resulted in an increase in diversity to the level observed in the control group, while the LE + D1 treatment exhibited the lowest diversity.

In contrast, the presence of additives and the duration of the experiment did not have any significant impact on bacterial diversity compared to fungal diversity (Fig. 8D). *Lysobacter* was initially abundant in both the control and D1 treatment on day 0, but its abundance decreased over time (Fig. S2A). However, this genus remained stable in the LE and LE + D1 treatments. *Pseudarthrobacter, Pontibacter, Planococcus*, and *Paracoccus* were initially abundant in the LE treatment, but their abundance significantly decreased on days 15 and 50. Both PC1 and PC2 were unable to account for the differences in treatment groups (Fig. S2B), indicating that additives have minimal influence on modifying the bacterial community composition.

## Discussion

*CA* was extensively cultivated in Yancheng City, Jiangsu Province, China. Concurrently, the presence of CCO was observed in *CA* plantations, prompting the implementation of traditional mitigation strategies such as soil flooding, fallow periods, and crop rotation. However, in order to optimize these efforts, it is imperative to comprehensively understand the underlying mechanisms. Notably, the proliferation of fungal pathogens emerged as a significant factor contributing to CCO incidence (Chen et al. 2012). In recent decades, various types of fungal pathogens have been identified through the use of both culturable and unculturable strategies in the context of CCO. For instance, during the CCO of American ginseng, *F. oxysporum* and *F. solani* were observed (Liu et al. 2020), while *Phoma eupyrena* and *F. culmorum* were prevalent in soil subjected to continuous wheat cropping (Bateman and Kwaśna 1999). Additionally, *Cylindrocarpon* sp. emerged as a prominent fungal pathogen in the continuous cropping of flue-cured tobacco (Wang et al. 2020). In this study, the primary fungal pathogen responsible for root rot disease in *CA*, specifically belonging to *F. solani*, was successfully isolated and confirmed. The three primers were utilized to verify the strain’s classification at the species level. While the TEF1 sequence primer did not yield as highly specific results as the ITS and RPB sequence primers, it was noted that TEF1 is commonly recommended for taxonomic identification of species within the genus *Fusarium* (O’Donnell et al. 2018). However, it is suggested that the ITS and RPB primers may be more suitable for identifying *F. solani*. As a prevalent pathogenic genus found in soil, *F. solani* is known to cause diseases in various crops such as melon (Ku et al. 2022), peanut (Oddino et al. 2008, Xie et al. 2017), soybeans (Ranzi et al. 2017), and so on. Our study demonstrates that the *F. solani* strain D1 can also act as an invasive pathogen, leading to root rot disease in *CA*.

When examining the stress response system in *CA*, most of the indices exhibited significant positive relationships, except for a negative correlation observed with POD activity (Table 1). This finding aligns with previous research on the subject (Boonsiri et al. 2007, Teixeira et al. 2017). Chl *a:b* ratio serves as an informative parameter for assessing the functionality of pigment equipment and the adaptability of the photosynthetic apparatus to varying light conditions (Lichtenthaler and Buschmann 2001). Moreover, it exhibits sensitivity toward environmental stressors such as drought (Ebrahimiyan et al. 2013), salt (Zhu et al. 2019), and high temperature stress (Bahrami et al. 2021). This study suggests that Chl *a:b* ratio can also be utilized as a reliable indicator to evaluate the stress status in response to leaf extract (LE) or root extract (RE). A Chl *a:b* ratio ranging from 0.6 to 0.8 is recommended for maintaining optimal health in plants.

**Table 1.** Pearson correlation coefficients among plant growth indices.

	Chl *a:b*	Chl *a*+*b*	Carotenoid	PAL	SOD	POD
Chl *a:b*	1.00	0.84**	0.76**	0.60**	0.86**	−0.36**
Chl *a*+*b*	0.84**	1.00	0.90**	0.84**	0.87**	−0.15
Carotenoid	0.76**	0.90**	1.00	0.78**	0.79**	−0.08
PAL	0.60**	0.84**	0.78**	1.00	0.81**	−0.12
SOD	0.86**	0.87**	0.79**	0.81**	1.00	−0.34*
POD	−0.36**	−0.15	−0.08	−0.12	−0.34*	1.00

* and ** indicate significant correlation between the corresponding data at 0.05 and 0.01 levels, respectively.

Numerous studies have indicated that the occurrence of CCOs can be attributed to shifts in microbial communities (Li et al. 2020, Chen et al. 2022b) and soil acidification (Bai et al. 2019, Li et al. 2021). Our research revealed that the continuous cropping of *CA* led to notable declines in pH over time (Fig. S1A), and the pH values in the LE and LE + D1 treatments exhibited significant decreases compared to the control (Fig. S1B). However, the magnitude of the decrease was <0.24 U, and the soil pH consistently remained above 7.4 throughout the 3-year continuous cropping process. Additionally, any temporary decline in pH swiftly rebounded to levels exceeding 7.0 (Fig. S1B). Therefore, based on the distinct primary factors contributing to CCO in various phases (Chen et al. 2020), it can be inferred that soil acidification may not be the primary underlying cause during this particular phase, as the observed changes in pH were not substantial and consistently remained above 7.0. Furthermore, our investigation did not reveal any significant alterations in bacterial diversity and community subsequent to the application of D1 and LE, which diverges from findings reported in previous studies (Bai et al. 2019, Liu et al. 2020). This discrepancy could potentially be attributed to variations in cropping duration and/or plant species. The most prominent shift in the fungal community was observed in the LE + D1 treatment. The introduction of LE had a significant impact on the fungal community over time, particularly evident in the increased abundance of *Fusarium*. This discovery suggests that LE has the potential to stimulate the growth and reproduction of strain D1, although the specific mechanism by which this occurs, whether through the provision of additional nutrients or the promotion of colonization, remains unclear. Furthermore, the presence of LE + D1 led to a suppression of fungal diversity, indicating that strain D1 was notably encouraged by LE and potentially able to occupy more ecological niches, which could be advantageous for colonization in the rhizosphere.

This study presents a novel hypothesis, supported by field investigation and previous research, suggesting that leaf extracts may have a significant impact on CCO. In our study, it was observed that the water extract derived from the leaf of *CA* exhibited greater inhibitory effects on seeds germination, seedlings growth, and soil enzymes activities in comparison to the RE. This finding contrasts with the known positive impacts of leaf litter on soil properties within forest ecosystems (Li et al. 2014) and other agricultural environments (Chen et al. 2022a). Limited research has been conducted on the identification of active compounds present in medicinal plant leaves that contribute to the development of tuberous roots. Our investigation revealed a higher accumulation of phenolic acids, specifically caffeic acid and *p*-coumaric acid, in the leaf tissue as opposed to the root tissue. Although the substances were found to be plentiful in both LE and RE, their degradation in soil occurred within a short span of a few hours (Bravetti et al. 2020), resulting in significantly lower concentrations in the soil (Fig. 7). Phenolic acids are widely recognized as the primary inhibitors in seed germination and plant growth processes (Lodhi 1975), with varying effects observed for different types of phenolic acids (Li et al. 2016b, Bravetti et al. 2020). While previous studies have frequently mentioned caffeic acid and *p*-coumaric acid, their specific interactions with the primary fungal pathogen and their impact on the microbial communities remain unclear. A recent study has identified three phenolic acids, namely chlorogenic acid, salicylic acid, and vanillic acid, as promoters of a greater relative abundance of soil-borne fungi capable of invading plant roots in a simulated rhizosphere (Clocchiatti et al. 2021). This finding aligns with another study that employed Illumina MiSeq sequencing technology (Li et al. 2018). Additionally, our research demonstrates that LE and RE can stimulate the growth of strain D1, primarily manifested through increased hypha weight and spore numbers (Fig. 5). The presence of thicker hyphae and a higher spore count in the soil undoubtedly enhances their competitive advantage within microbial communities. Although researchers have suggested that certain extracts from medicinal plants may have an antagonistic effect on *Fusarium* spp. (Dwivedi and Yadav 2015), our findings partially align with another study that demonstrated the ability of pathogenic *Fusarium* spp. to utilize ginsenoside, an active compound (Jiao et al. 2015). This discrepancy could be attributed to the use of different substances in these studies. It is worth noting that LE or RE also induced the stimulation of certain pathogenesis-related enzymes (Fig. 6), which might contribute to the heightened negative effects on the growth of *CA* seedlings when invaded by strain D1 in conjunction with LE or RE (Fig. 6). This phenomenon may serve as a significant factor contributing to the heightened detrimental effects on the growth of *CA* seedlings subsequent to the invasion of strain D1 in conjunction with LE or RE. This finding aligns with prior research, as evidenced by a separate study which demonstrated that the fungus predominantly exhibited extracellular biodegradative enzymes (such as proteases, pectinases, and cellulases) in cultures containing litter (Schneider et al. 2010). Additionally, another investigation indicated that the application of agave leaf extracts could stimulate enzyme activities in fungal strains belonging to *Fusarium* and *Lasiodiplodia* (Campos‐Rivero et al. 2019). Given that phenolic acids in leaves have the potential to induce the growth and proliferation of strain D1, it is reasonable to comprehend the subsequent bloom of D1 in the leaf litter treatment during a later phase (Fig. 4D).

## Conclusions

In conclusion, the mechanisms underlying CCO induced by LE or RE encompass the modulation of seed germination and plant growth, suppression of the plant’s immune response, augmentation of pathogens, as well as the stimulation of fungal pathogen strain D1 growth and inhibition of plant pathogenesis-related enzyme activities. These effects are potentially attributed to the presence of caffeic acid and *p*-coumaric acid, particularly in LE. Consequently, it is advisable to discontinue the practice of disposing leaf litters in soil, instead opting to gather them for potential resource utilization, such as weed control, extraction of active compounds, and formulation of functional animal feeds in forthcoming endeavors.

## Supplementary Material

fiae068_Supplemental_File
