# Supplementary material for: Leaf litter from Cynanchum auriculatum Royle ex Wight leads to root rot outbreaks by Fusarium solani, hindering continuous cropping
Source: FEMS Microbiol Ecol. 2024 Apr 29;100(6):fiae068. doi: 10.1093/femsec/fiae068 (PMC11099666; doi:10.1093/femsec/fiae068)
Supplement: fiae068_Supplemental_File [file fiae068_supplemental_file.docx]

**Supplementary Materials**

**Title**

Leaf litter from Cynanchum auriculatum Royle ex Wight leads to root rot outbreaks by Fusarium solani, hindering continuous cropping

**Authors**

Min Shen ^abc^, Limeng Wu ^b^, Yanzhou Zhang ^b^, Ruiqiang You ^b^, Jiaxin Xiao ^a^, Yijun Kang ^b,c*^

*^a^ College of Life Sciences, Anhui Normal University, Wuhu, Anhui, China*

*^b^ Jiangsu Key Laboratory for Bioresources of Saline Soils, Yancheng Teachers University, Yancheng, Jiangsu, China*

*^c^ Jiangsu Key Laboratory for Bioresources of Saline Soils, Jiangsu Provincial Key Laboratory of Coastal Wetland Bioresources and Environmental Protection, Yancheng Teachers University, Yancheng, China*

**Running title**

Leaf litter contributes to CCO

**Corresponding author**

Yijun Kang

Mailing address: 2 South Hope Avenue, Yancheng Teachers University, Yancheng, Jiangsu, P. R. China, 224007

E-mail: [yjkang@yctu.edu.cn](mailto:yjkang@yctu.edu.cn)

Tel/Fax: +86 (0) 515 88233660

Fig. S1

| 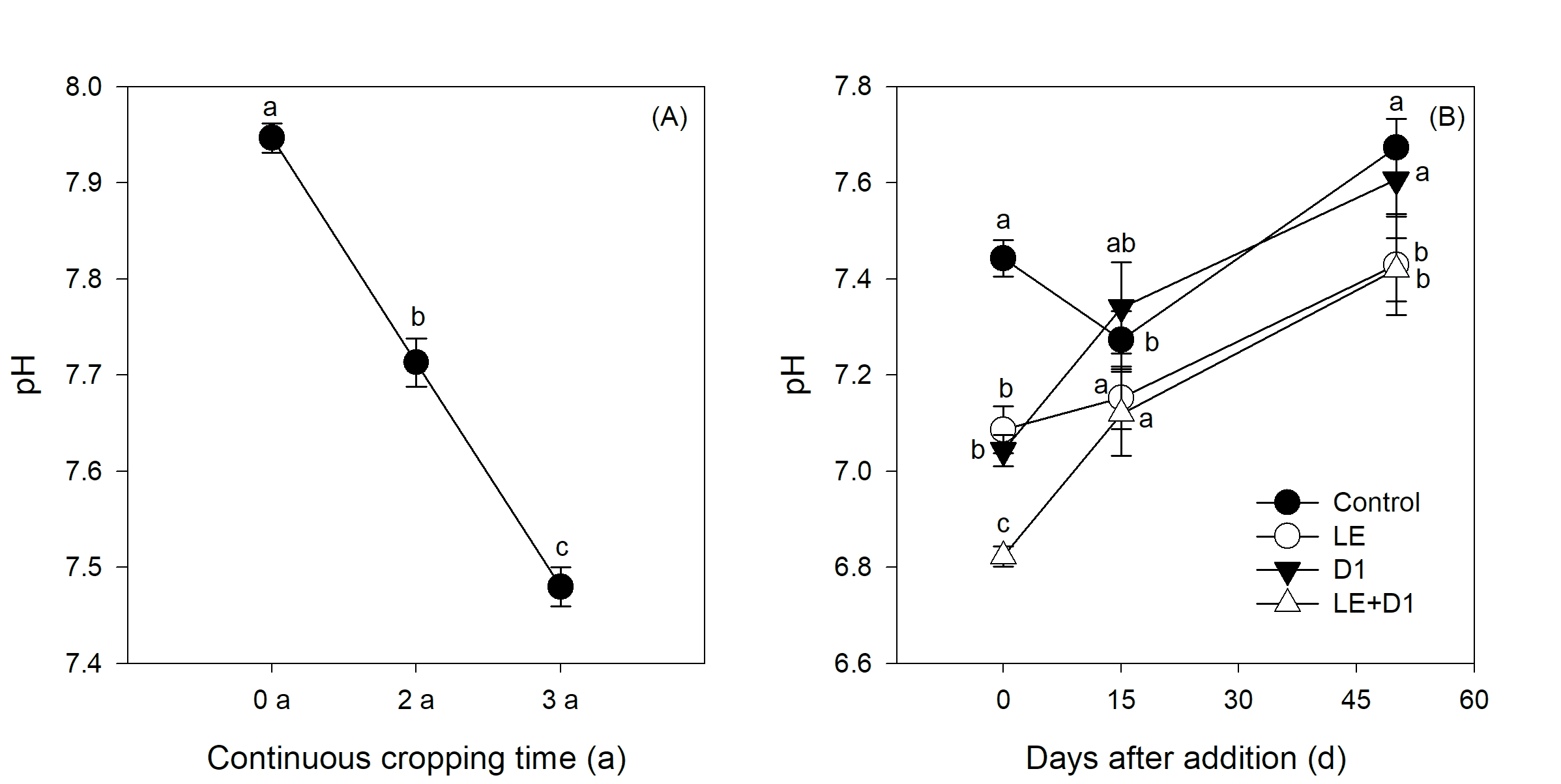 |
| --- |

Fig. S2


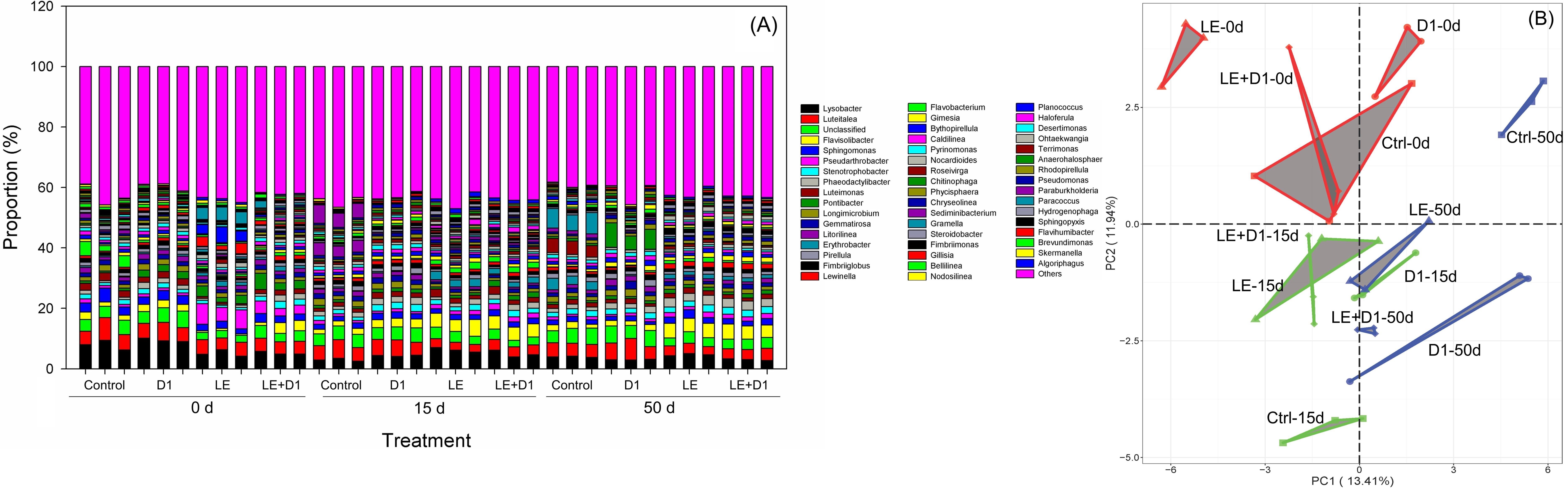


Fig. S3


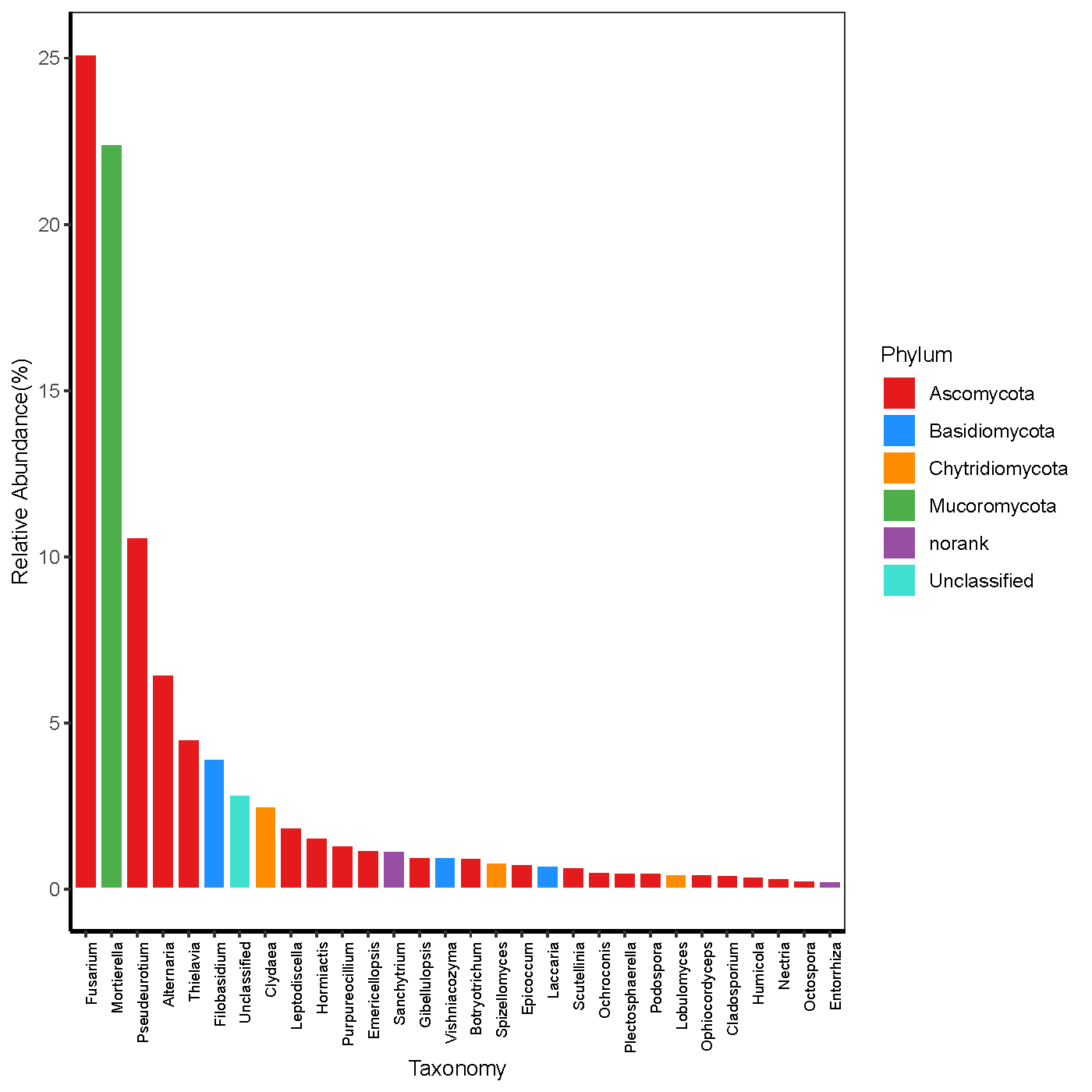


**Figure legends:**

Fig. S1 Changes in pH of soils planting of *CA* with different continuous cropping histories (A), and comparisons of soil pH with different treatments (B). 0 a, 2 a, and 3 a indicated that soils have been planted with *CA* for 0, 2, and 3 years, respectively. Different letters at one sampling time indicates significant differences among treatments (Duncan’s multiple range test, P≤0.05).

Fig. S2 Effects of LE and/or strain D1 on the bacterial community with time (A), and the changes in bacterial composition among different treatments during 50 days using Principal component analysis (PCA) (B). 0d represents the initial bacterial community, 15d and 50d represents day 15 and day 50, respectively. Those relative abundance lower than 1% were categorized to “others”.

Fig. S3 The top 30 dominant taxonomies at the genus level. Different colors represent different phylum for each genus.
